# Technological and probiotic potential of BGRA43 a natural isolate of *Lactobacillus helveticus*

**DOI:** 10.3389/fmicb.2013.00002

**Published:** 2013-01-23

**Authors:** Ivana Strahinic, Jelena Lozo, Amarela Terzic-Vidojevic, Djordje Fira, Milan Kojic, Natasa Golic, Jelena Begovic, Ljubisa Topisirovic

**Affiliations:** ^1^Institute of Molecular Genetics and Genetic Engineering, University of BelgradeBelgrade, Serbia; ^2^Faculty of Biology, University of BelgradeBelgrade, Serbia

**Keywords:** *Lactobacillus helveticus*, milk fermentation, probiotics, prebiotics

## Abstract

*Lactobacillus helveticus* BGRA43 is a human intestinal isolate showing antimicrobial activity, amongst others, against *Yersinia enterocolitica*, *Shigella sonnei*, *Shigella flexneri*, and *Streptococcus pneumoniae*. BGRA43 produces PrtH proteinase with proteolytic activity on both casein and β-lactoglobulin (BLG). BGRA43 is able to reduce the allergenicity of BLG. Bioactive peptides released in BGRA43 fermented milk are potent modulators of innate immunity by modulating the production of proinflammatory cytokines IL-6 and TNF-α. BGRA43 is able to survive in simulated gastric and intestinal conditions. The growth of BGRA43 in milk results in a fast acidification lowering the milk pH to 4.53 generating mild, homogeneous, and viscous yogurt-like product. The strain BGRA43 grows suitably in pure cow or goat’s milk as well as in milk containing inulin or nutrim even when they are used as the sole carbon source. It is suggested that strain BGRA43 could be used as a single-strain culture for the preparation of yogurt-like products from bovine or caprine milk. Overall, *L. helveticus* BGRA43 could be considered as a potential probiotic candidate with appropriate technological properties attractive for the dairy industry.

## INTRODUCTION

*Lactobacillus helveticus* belongs to a homofermentative *L. acidophilus/L. delbrueckii* subgroup of lactobacilli (Felis and Dellaglio, 2007) associated to fermented milk, meat, and plant products as well as the gastrointestinal (GI) and urogenital (UG) tracts of humans and animals. *L. helveticus* has been traditionally used as a starter in the production of Italian and Swiss-type cheeses (Giraffa et al., 1998; Beresford et al., 2001). In the last 10 years, the scientific knowledge about different *L. helveticus* strains with health-promoting properties have accumulated (de Moreno de LeBlanc et al., 2005; Guglielmetti et al., 2010; Ohland and MacNaughton, 2010; Slattery et al., 2010). Significant features that provide for the broad use of *L. helveticus* in food and feed biotechnology are often complemented with demonstrable probiotic characteristics such as antimicrobial, immunomodulatory, and antihypertensive activities (LeBlanc et al., 2004; Atassi et al., 2006; Fitzgerald and Murray, 2006). In order to identify the key genes and mechanisms contributing to the technological properties and interactions with a host, complete genome sequencing of different *L. helveticus* strains has been performed. Moreover, detailed functional assays implicate the potential nutritional and health benefits of products containing *L. helveticus* strains (Callanan et al., 2008; Prajapati et al., 2011; Zhao et al., 2011).

## CHARACTERIZATION OF THE *L. helveticus* BGRA43

The strain *L. helveticus* BGRA43 is a natural isolate originating from the intestinal tract of healthy man. The strain was isolated by classical microbiological methods, and previously characterized as *L. acidophilus* only according to carbohydrate fermentation pattern (Banina et al., 1998). Later on, the strain was reclassified and phylogenetically identified by complete 16S rDNA sequencing, combined with AFLP, PFGE, and SDS analyses, as *L. helveticus*, and deposited in the Belgian Coordinated Collections of Microorganisms (BCCM), Gent, Belgium, as LMG P-24226 (Lozo et al., 2011). The general characteristics of BGRA43 are as follows: (a) growth is optimal under anaerobic conditions in the presence of 10% CO_2_; (b) it grows in the presence of 2% NaCl, but not in the presence of either 4 or 6.5% NaCl; (c) during its growth in milk, it forms a homogeneous curd showing extensive auto-aggregation giving the curd a slimy appearance, and (d) it efficiently hydrolyzes lactose. The BGRA43 strain grows well in MRS broth at 37 and 45°C after successive sub-cultivations but not at 15°C. The optimal growth conditions for the BGRA43 strain have been determined and results show that the shortest generation time can be achieved when the cells are grown in MRS broth at 37°C. The stationary growth phase is reached after 12 h. However, it was shown that during its growth at 42°C, the log period is longer and the stationary growth phase is attained after 14 h, although at this temperature the total cell number is slightly higher compared to growth at 37°C.

A detailed characterization of the BGRA43 strain was also performed, including tests of its antimicrobial and proteolytic activity, as well as a study of its probiotic and technological properties. The results revealed that BGRA43 exhibits significant antimicrobial activity against a variety of microorganisms, including *Staphylococcus aureus*, *Escherichia coli* C600, *Bacillus mycoides*, *Pseudomonas* sp., *Yersinia enterocolitica*, *Shigella sonnei*, and *Shigella flexneri*, as well as *Streptococcus pneumoniae*. In addition, the experiment performed in sulfite agar showed that BGRA43 exhibited an inhibitory effect on the growth of *Clostridium sporogenes*, widely distributed in nature as a food contaminant, as well as a human and animal pathogen. It was found that any growth of clostridia could not be detected in co-culture with BGRA43 (Banina et al., 1998). Additionally, the nature of the BGRA43 antimicrobial activity was tested using fresh bacterial cultures (grown for 9, 12, and 16 h) from which cell-free and neutralized cell-free filtrates were prepared and examined in parallel. The possibility that the compound possessing an antimicrobial activity is of a proteinaceous nature was excluded, since in the well-diffusion assay for detection of bacteriocins the characteristic reduction of the inhibitory zone of indicator strain growth was absent after the addition of the proteolytic enzymes – pronase E, proteinase K, trypsin, and pepsin (Strahinic et al., 2012).

While combination of lactic and acetic acids can reduce bacterial growth, lower degree of dissociation of these acids (under pH 4–4.6) might be associated with strong antimicrobial activity (Ouwehand, 1998). Inhibitory effect of BGRA43 is completely lost when supernatant was used instead of fresh bacterial culture. These results suggested that organic acid/s are involved in antimicrobial activity of BGRA43. We can only speculate that synergistic effect of lactic and acetic acid in combination with some currently unknown compounds provided antimicrobial activity to a wide range of bacteria.

It is well known that the proteolytic activity of lactobacilli, in terms of the degradation of casein fractions, plays an important role in the development of texture and flavor during the fermentation process and is very often essential for optimal growth in milk. Well-defined proteinases of lactobacilli are the cell-envelope proteinases (CEP) that possess different domain organization. The characterization of the proteolytic activity of *L. helveticus* BGRA43 included the analysis of α_s1_-, β-, κ- and total-casein degradation. Additionally, the gene(s) encoding for CEP present in the BGRA43 genome were determined. The results revealed that the whole cells of BGRA43 possessed a strong caseinolytic activity. It was shown that BGRA43 was able of completely hydrolyzing α_s1_- and β-casein fractions after 1 h of incubation in 100 mmol Na-phosphate buffer, pH 6.5 at 45°C. The whole cells also hydrolyzed κ-casein under the same conditions but after 2 h of incubation. The obtained results suggested that the release of the proteinase from the cell envelope of BGRA43 is not Ca^2+^ dependent. The activity of the crude proteinase extract was partially inhibited in the presence of Cu^2+^ and Zn^2+^ ions, but also by EDTA, EGTA as well as PMSF (Fira et al., 2001).

From a genetic point of view the organization of genes encoding CEP in the BGRA43 strain appears to be different from all *L. helveticus* strains described so far. Until now, two CEPs have been described and completely analyzed in *L. helveticus*, PrtH2 and PrtH. Both belong to the subtilisin-like serine proteinase family with whom they share 22% amino acid sequence identity. According to the work of Genay et al. (2009), the *prtH2* gene is a ubiquitous gene in *L. helveticus*, and it is present in all tested strains, whereas the presence of the *prtH* gene is strain-dependent. In our study, PCR analysis with primers corresponding to the specific regions of the *prtH* and *prtH2* genes both present in *L. helveticus* CNRZ32, showed that BGRA43 possesses only the *prtH* gene, sharing 98.9% of identity with the same gene of CNRZ32. The hypothesis that PrtH is most probably the only active proteinase in the strain BGRA43 was further confirmed after obtaining the proteolytically inactive derivative of BGRA43 designated BGRA433. Results showed that the derivative BGRA433 carries a deletion within the *prtH* gene encoding the catalytic region of the proteinase (Lozo et al., 2011). Recently, a study on distribution of CEP paralogs among different strains of *L. helveticus* revealed that the most common CEP gene is *prtH3*, compared to the *prtH*, *prtH2*, and *prtH4* (Broadbent et al., 2011). Only one strain tested in this study has only *prtH* gene, but its proteolytic activity, biochemical characterization and other features were not analyzed.

The fact that the strain BGRA43 possesses only the PrtH proteinase, in contrast to other tested *L. helveticus* strains, could be especially interesting in relation to the production of the strain-specific bioactive peptides as a result of casein hydrolysis. Mass spectrometry analyses of the peptides released after β-casein hydrolysis by BGRA43 identified 22 peptides, varying from 5 to 46 amino acids. The uniqueness of the BGRA43 strain is reflected in the observation that the released peptides originated from the more hydrophilic N-terminus portion of β-casein, as well as that the numerous cleavages of β-casein occurred after phenylalanine. The peptides identified after 24 h of β-lactoglobulin (BLG) hydrolysis varied from 5 to 17 amino acids; most of them had a hydrophobic amino acid on their C-terminus. Interestingly, the obtained results show that one of the three major allergenic epitopes of BLG at the Val^41^, Tyr^42^, and Val^43^ positions is hydrolyzed by the BGRA43 crude extract (Lozo et al., 2011; Strahinic et al., 2012). Moreover, four protein fractions released during BGRA43 milk fermentation exhibited an inhibitory effect on human monocyte RB, as well as anti-inflammatory effects on the Nd-THP-1 monocyte cell line due to the down-regulation of TNF-α or IL-6 production (Tompa et al., 2010).

According to the FAO/WHO guidelines (FAO/WHO, 2006), the main criterion for probiotic selection and application is the survival of the bacterial cells during their passage through the GI tract. The strain BGRA43 was examined in a chemically simulated GIT transit, which included an assessment of its survival in gastric and intestinal juices. The obtained results show that *L. helveticus* BGRA43 survived GIT passage much better when it was included in a food matrix (skimmed milk), probably due to the buffering and protective effect of the skimmed milk. While the strain could not survive the simulated GIT conditions in a saline buffer, the survival of BGRA43 in simulated gastric juice supplemented with skimmed milk during 90 min of incubation was 100%. The first short exposure to bile salts (10 min, 0.6%) reduced the bacterial viability by three log units, but after lowering the concentration of bile salts to 0.3%, the number of surviving bacteria was higher (Strahinic et al., 2012).

To evaluate the hydrophobic/hydrophilic cell surface properties of *L. helveticus* BGRA43, the affinity of the strain to the organic solvents hexadecane and chloroform was tested. Preliminary results pointed out a high affinity for both 70% hexadecane and 75% chloroform which is in agreement with values obtained for other human isolates (Ocana et al., 1999).

**FIGURE 1:**
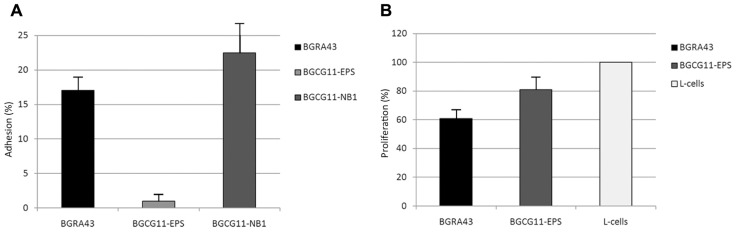
**Mean values of the adhesion (%) of *Lactobacillus helveticus* BGRA43 and control strains *Lactobacillus paraplantarum* BGCG11 and its non-ropy derivative BGCG11-NB1 to the human epithelial intestinal Caco-2 cell line**. The statistical difference (*p* < 0.5) between BGRA43 and BGCG11 was determined by Student’s *t*-test **(A)**. Proliferation (%) of GALT isolated from the rat was co-cultured for 4 days in the presence of UV-irradiated *Lactobacillus helveticus* BGRA43 and the control strain *Lactobacillus paraplantarum* BGCG11 at a bacteria : cell line ratio of 5:1. The statistical differences (*p* < 0.05) between each stimuli and the control (L-cells: GALT lymphocytes maintained in RPMI-medium) were determined by Student’s *t*-test **(B)**.

Another criterion provided by the FAO/WHO guidelines for probiotic evaluation is the ability of a strain to colonize transiently the intestinal mucosa. Therefore, the adhesion ability of *L. helveticus* BGRA43 to the Caco-2 epithelial intestinal cell line was determined. As controls, the exopolysaccharide (EPS)-producing strain *L. paraplantarum* BGCG11 and its non-ropy derivative BGCG11-NB1 were used (Nikolic et al., 2012). The BGCG11 strain, as was shown previously, has an adherence property similar to the well-known probiotic strain *L. rhamnosus* GG. The derivative BGCG11-NB1 showed significantly higher adherence to epithelial intestinal cell lines compared to GG and BGCG11 strains. It seems that the EPS synthesized by BGCG11 strain covers the producing strain and hinder bacterial adhesion to enterocytes whereas in the absence of EPS other surface molecules could have been exposed and therefore act as adhesins. In general, the adhesion of BGRA43 to the Caco-2 cell line was similar to the adherence of the derivative BGCG11-NB1, and significantly higher (*p* < 0.05) than the adherence of BGCG11 (**Figure 1A**). These findings indicated that the BGRA43 strain has a significantly better adherence trait than the probiotic and reference strain *L. rhamnosus* GG.

The capability of the *L. helveticus* BGRA43 strain inactivated by UV-radiation to elicit the immune response was tested by examining the induction of proliferation of gut-associated lymphoid tissue (GALT) isolated from rats. The proliferation indices of GALT measured in the presence of BGRA43 and the *L. paraplantarum* BGCG11 strain, which served as a positive control, show that in the absence of stimulus the presence of both strains significantly (*p* < 0.05) decreased the proliferation of GALT in comparison with the control (**Figure 1B**). The results show that the strain BGRA43 has a strong immunomodulatory effect, revealed as a reduction in the proliferation of lymphocytes, and that it could play a role in the suppression of the immune response *in vivo*. Taking into account that the immunomodulatory ability of probiotics is not effective in all human populations it could be assumed that the strain BGRA43, which possesses a potential immunosuppressive effect, could be specifically used in a diet of autoimmune disorder patients that present an increased inflammatory status. However, according FAO/WHO and EFSA recommendations, before strains such as BGRA43 can be used for human consumption the strain needs to be tested in human clinical trials.

## TECHNOLOGICAL PROPERTIES OF *L. helveticus* BGRA43

*Lactobacillus helveticus* strains are traditionally used for the production of different types of cheeses primarily for long-ripening cheeses such as Parmesan, Emmental, Grana, Provolone, and Gruyere (Torriani et al., 1994; Giraffa et al., 1998; Giraffa and Neviani, 1999; Rossetti et al., 2008). During our work, we tried to apply BGRA43 as a starter in dairy fermentation to obtain a high-quality yogurt-like product.

The ability of BGRA43 to grow and curdle milk was initially tested in reconstituted skim milk (RSM). *L. helveticus* BGRA43 exhibited rapid growth at 37°C and 42°C in RSM; after 6 h of growth the bacterial culture reached approximately 3 × 10^8^ CFU/ml (Banina et al., 1998). The growth of BGRA43 in a chemically defined medium (CDM) containing either inulin or nutrim as carbohydrate source, was tested in parallel. These two fructans are dietary fibers exploited as potential prebiotic stimulators of intestinal bacterial growth. BGRA43 displayed very satisfactory growth in the presence of either inulin or nutrim, even when they were the sole carbon source. The total cell number was approximately 10^7^ CFU/ml and was comparable to the cell number obtained when the monosaccharides, glucose and lactose, were used in CDM as a carbon source.

The fermentation ability of BGRA43 in various milk sources on a laboratory scale was examined. For this purpose, aside from cultivation by RSM, commercial UHT sterilized whole cow’s milk (WCM), obtained from a local supermarket and whole goat’s milk (WGM), obtained from a local farm, were used. Furthermore, the fermentation ability of BGRA43 in RSM, WCM, and WGM containing inulin (1%) or nutrim (1%) was also tested. At the end of a 4 h fermentation at 42°C, the number of viable cells in each of the nine milk combinations increased from 10^6^ to 10^8^ CFU/ml, while the pH values ranged between 4.35 and 4.69, depending on the milk. At the same time, the titratable acidity of the obtained yogurt-like products was measured. Acidity varied among the products. Thus, at the end of fermentation, acidity did not exceed 27.6°SH, 39.2°SH, and 29.6°SH for products obtained in RSM, WCM, and WGM, respectively. Similar results were obtained for the remaining fermented products containing inulin or nutrim. It is worthwhile mentioning that the highest viscosity was observed in yogurt-like products prepared with either inulin or nutrim. This trend of viscosity was maintained even after 7 days of storage at 4°C. During the same storage conditions, the viable count of BGRA43 was 10^6^ CFU/ml in all of the tested product samples. These properties lend further support for the use of BGRA43 as a starter culture strain. Finally, after 7 days of storage, the yogurt-like products made from RSM, WCM, and WGM, all of which contained 1% inulin, retained sufficient acidity and received high scores by sensory analysts, which was not the case when nutrim was present (Strahinic et al., 2012). A test on yogurt-like production using BGRA43 as starter under industrial conditions was performed in one private SME. Obtained product showed almost the same sensory characteristics as that produced on laboratory scale.

## CONCLUSION

In this mini-review, we present the key features of human intestinal isolate *L. helveticus* BGRA43. This isolate is potential probiotic strain that can find application in dairy industry. The optimal growth of this auto-aggregating strain is between 37 and 42°C in either MRS medium or skimmed milk. During its growth, BGRA43 cells show strong antimicrobial potential against various sporogenic and pathogenic bacteria, probably due to rapid increase of organic acids in the growth medium. Under optimal conditions, PrtH proteinase from BGRA43 completely hydrolyze all three casein fractions. In addition, BGRA43 cells are able to hydrolyze BLG and reduce its allergenicity, thus contributing to better digestibility – especially in people allergic to cow’s milk. BGRA43 cells are sensitive to gastric and intestinal conditions, but when applied in skimmed milk that serves as a protector, they survive simulated digestion. More than 70% of BGRA43 bacteria adhere to non-polar solvents, hexadecane and chloroform, classifying it as a high hydrophobic strain. Strong adhesion ability of BGRA43 to intestinal epithelial cell lines was subsequently confirmed *in vitro* with Caco-2 cell line.

Four oligopeptides showing anti-inflammatory effect were detected after BGRA43 milk fermentation. Moreover, reduction of lymphocytes proliferation confirmed that BGRA43 cells possess strong immunosuppressive potential.

Finally, technological properties of this strain were also satisfactory when tested on a laboratory and small industrial scale. However, for application as starter culture in production of fermented milk products, further experiments on a larger industrial scale are necessary.

## Conflict of Interest Statement

The authors declare that the research was conducted in the absence of any commercial or financial relationships that could be construed as a potential conflict of interest.

## Acknowledgments

The authors are grateful to Ms Myra Macpherson-Poznanovic, and Dr. Goran Poznanovic for editing the text. The Ministry of Education, Science and Technological Development of the Republic of Serbia, Grant No. 173019, supported this work.
